# Assessment of left ventricular twist mechanics by speckle tracking echocardiography reveals association between LV twist and myocardial fibrosis in patients with hypertrophic cardiomyopathy

**DOI:** 10.1007/s10554-014-0509-6

**Published:** 2014-08-09

**Authors:** Hong-Ju Zhang, Hao Wang, Tao Sun, Min-Jie Lu, Nan Xu, Wei-Chun Wu, Xin Sun, Wu-Gang Wang, Qiong-Wen Lin

**Affiliations:** 1 Department of Ultrasound, State Key Laboratory of Cardiovascular Disease, Fuwai Hospital, National Center for Cardiovascular Diseases, Chinese Academy of Medical Sciences and Peking Union Medical College, No. 167, Bei Lishi Road, Xicheng District, Beijing, 100037 People’s Republic of China; 2 Division of Cardiology, Beijing Anzhen Hospital, Capital Medical University, Beijing, 100029 People’s Republic of China

**Keywords:** Hypertrophic cardiomyopathy, Cardiac magnetic resonance, Speckle tracking echocardiography, Myocardial fibrosis

## Abstract

We aimed to investigate whether left ventricular (LV) twist analysis can detect the extent of myocardial fibrosis in patients with hypertrophic cardiomyopathy (HCM). This prospective case–control study recruited 81 consecutive patients with HCM examined between January 2012 and April 2013. Data of 76 patients were analyzed after excluding 5 patients whose echocardiographic images were of poor quality. Healthy volunteers (n = 46) served as controls. Both groups underwent comprehensive echocardiographic examination (i.e., Bas-Rotation, AP-Rotation, LVEF, LADs, IVST, LAVi, E/Em, LVMI, advanced LV-twist analysis by speckle tracking echocardiography) and magnetic resonance imaging. Between-group differences were analyzed by independent *t* test; logistic regression analysis was performed to identify effect factors. No significant differences were found between baseline characteristics of HCM and control groups (all *p* > 0.05). HCM patients had significantly higher Bas-Rotation, AP-Rotation, LV Twist, LVEF, LADs, IVST, LAVi, E/Em and LVMI than controls (all *p* < 0.0001) and significantly lower LVDd and E/A (both *p* < 0.001). Bas-Rotation, AP-Rotation, LV-Twist, LADs, IVST, LAVi, E/Em and LVMI were significantly higher in HCM patients with fibrosis than in those without fibrosis (*p* < 0.001), but no significant differences in other echocardiographic parameters were found between those with and without fibrosis. Age, Bas-Rotation, AP-Rotation, LV twist, LADs, IVST, LAVi, E/A, E/Em, and LVMI were significant effect factors for fibrosis. AUROC analysis showed that LV twist had high discriminatory power to detect extent of myocardial fibrosis (AUC 0.996, 95 % CI 0.989–1.004, *p* < 0.001). Left ventricular twist mechanics are associated with the extent of myocardial fibrosis. LV-twist assessment by STE may be clinically useful.

**Electronic supplementary material:**

The online version of this article (doi:10.1007/s10554-014-0509-6) contains supplementary material, which is available to authorized users.

## Introduction

Myocardial fibrosis is a pathological entity associated with extracellular matrix remodeling, which may lead to increased myocardial stiffness and left ventricular systolic and diastolic dysfunction [1, 2]. Late gadolinium enhancement (LGE) by cardiovascular magnetic resonance imaging (CMRI) is frequently observed in patients with hypertrophic cardiomyopathy (HCM) and allows in vivo quantification of myocardial fibrosis [3, 4]. Previous studies in large HCM cohorts have noted that delayed enhancement by CMRI such as LGE is associated with left ventricular (LV) systolic and diastolic dysfunction, including increased risk of non-sustained ventricular tachycardia (NSVD) that may lead to adverse cardiac events such as sudden cardiac death, fatal arrhythmia, or worsening heart failure in HCM patients [3, 4]. Thus, it becomes important to identify HCM patients at higher risk for ventricular arrhythmias and sudden cardiac death [1]. However, although contrast-enhanced cardiovascular MRI like LGE is able to identify the underlying abnormal myocardial substrate that comprises fibrosis [3], the clinical significance of these findings is still not well understood [4]. Besides the prognostic value of delayed contrast enhancement in CMRI [5], evaluation of other parameters may be needed to accurately evaluate risk in HCM patients.

Speckle tracking echocardiography (STE), which is based on tracking and measurement of tissue displacement, has the potential to accurately and reliably assess myocardial mechanics, providing a relatively simple, noninvasive approach to the study of LV rotation and twist [6–9]. LV twist represents the mean longitudinal gradient of the difference in clockwise and counter-clockwise rotation of the left ventricular apex and base, a phenomenon that links systolic contraction with diastolic relaxation and plays a major role in cardiac physiology [10]. Choudhury et al. [11] reported that regional myocardial strain and wall thickening were affected by regional myocardial fibrosis in patients with HCM. LV rotation occurs when myocardial fibers contract and, as such, impaired LV twist mechanics may be the basis of LV dysfunction in the presence of acute increase in afterload [12]. However, the association between LV twist and the extent of myocardial fibrosis is unknown, and the clinical significance of LV twist mechanics remains unclear in HCM patients. To the best of our knowledge, studies examining the relationship between LV twist mechanics and the extent of myocardial fibrosis have not been published. We hypothesized that the presence of greater myocardial twist may be associated with a greater degree of myocardial fibrosis in patients with HCM and that LV twist mechanics assessment may help differentiate the presence of fibrosis. We expected that the relationship between LV twist and myocardial fibrosis could be confirmed by using STE in conjunction with other echocardiographic parameters and MRI imaging. Therefore, the aim of this study was to investigate whether assessment of LV twist mechanics by STE is able to detect the extent of myocardial fibrosis and serve as a novel prognostic parameter in HCM patients.

## Methods

### Patients

This prospective case–control study recruited a total of 81 consecutive patients with HCM who were examined in our department between January 2012 and April 2013. The diagnosis of HCM was made by echocardiography based on the criteria of the World Health Organization/International Society and Federation of Cardiology. All patients had also undergone MRI examination. Patients’ medical records were thoroughly reviewed to ensure that there was no history of hypertension, and no patient presented with hypertension during the study period. Patients were excluded if they had poor resolution of echocardiographic images; obstructive HCM (LV outflow tract gradients >30 mmHg under basal conditions or after Valsalva maneuver); left ventricular ejection fraction (LVEF) <50 % as assessed by echocardiography; cardiac muscle disease secondary to any known systemic condition; atrial fibrillation, implantation of a pacemaker or defibrillator; significant valvular heart disease; or known coronary artery disease (lesions >50 % on angiogram). Finally, after five patients were excluded because their echocardiographic images were of poor quality and not suitable for the present study, the data from 76 patients were retained for subsequent analysis.

Patients were classified as having asymmetric septal hypertrophy if wall thickness in the basal or mid anterior/inferior septum was >1.3 cm and septal/lateral wall thickness ratio was >1.3. Patients were classified as having a septal bulge if the proximal anterior/inferior septum was >1.3 cm and the mid or apical anterior/inferior septum was <1.3 cm. Apical hypertrophy was diagnosed if the ratio of maximal segmental wall thickness of the apex and the rest of the ventricle was >1.5. All patients were in sinus rhythm.

All drugs were discontinued at least 24 h before echocardiography and MRI evaluation, as previously recommended [13]. Patient history of syncope (transient and complete loss of consciousness) and family history of sudden cardiac death in a first-degree relative were recorded.

For the purpose of analysis, the HCM group (n = 76) was further divided (after imaging and echocardiographic examinations described below) into a fibrosis group and a non-fibrosis group.

In addition, a group of 46 healthy volunteers who underwent advanced LV-twist analysis by echocardiography served as controls. These subjects had no documented history of cardiovascular disease, including hypertension or HCM. These subjects did not undergo MRI evaluation.

### Ethical considerations

This study was reviewed and approved by the hospital’s Institutional Review Board. All included patients and control subjects provided signed informed consent.

### MRI examination and image analysis

Magnetic resonance imaging examination was performed within 2 weeks of echocardiographic examination. MRI was performed using a 1.5-T Sonata scanner (Siemens Corporation, Erlangen, Germany). Electro cardio-gating breath-holding sequential scanning was carried out with the patient in the dorsal position. Axial and sagittal single-shot turbo spin echo imaging was carried out on the basis of conventional axial, sagittal and coronal phases. Cine-imaging was carried out for left ventricular two-chamber and four-chamber longitudinal planes as well as LV outflow tract planes and LV shot-axis view for 6–8 layers, and the scanning sequence was retrospective ECG gating true fast imaging, as described previously [14]. The left ventricular mass at end-diastole was semi-automatically calculated on the basis of Simpson’s rule, using a work station and manual revision by visual assessment. The left ventricular mass was normalized to body surface area. Images for LGE diagnostics were acquired 10 min after the injection of gadopentetate dimeglumine 0.2 mmol/kg (Magnevist; Schering Corporation, Berlin, Germany) with breath-hold segmented inversion-recovery sequences acquired in the same view. Myocardial perfusion and delayed scanning were analyzed according to the 17-segment method for the left ventricle recommended by the American Heart Association (AHA) [15]. The presence of late enhancement within each segment was assessed semiquantitatively using a 17-segment model of the LV [16]. LGE of the LV was considered present if the signal intensity of hyperenhanced myocardium was >5 standard deviations above the mean signal intensity of vital myocardium. MRI analysis was completely blinded from the echocardiographic and clinical analysis.

### Echocardiography examination

Echocardiography was carried out using the Phillips iE33 Color Doppler Ultrasound System with probe frequency 3.5 MHz (Philips Healthcare, Andover, MA, USA). The Qlab multiparameter analysis workstation (Phillips) was equipped with STE analysis software. The second-harmonic B-mode images of apical (4-chamber, 2-chamber) and short-axis (at the mitral valve and apical level) views were obtained. The frame rate was more than 50 frames/s. The LV endocardial border was manually traced at the end-systolic frame and the speckle tracking region of interest was automatically selected. The width of the region of interest was adjusted as necessary to accommodate the total thickness of the LV wall [17]. The computer automatically tracked stable objects in each frame using the sum of absolute differences algorithm. After these steps, the work station computed and generated strain curves, as previously described [18].

The peak values of basal rotation were defined as the maximum negative values of the curves from the short-axis view at the mitral valve level. The peak values of apical rotation were defined as the maximum positive values of the curves from the short-axis view at the apical level [19]. Every view considered was divided into six segments, giving six different values from which mean values were determined. LV twist was defined as the difference between the mean values of the peak rotation at the apical and mitral valve level (twist = mean peak apical rotation − mean peak basal rotation) [10].

Standard two-dimensional measurements were obtained using standard methods. LVEF was calculated using the two-dimensional Simpson’s biplane method. LV mass was assessed with the two-dimensional area-length method and the left ventricular mass was normalized to body surface area, as previously described [20]. Left ventricular diameter in end-diastolic (LVDd), inter-ventricular septal thickness (IVST), left ventricular posterior wall thickness (LVPWT), and relative wall thickness (RWT) were measured according to criteria of the American Society of Echocardiography. Left atrium end-systolic dimensions (LADs) were measured at end-systolic, and LA volume was calculated using the prolate ellipsoid model. The LA volume index (LAVi) was calculated as LA volume divided by body surface area, reported as milliliters per square meter. The following variables were measured at the tips of the mitral valve leaflets from the apical 4-chamber view using pulsed-wave Doppler: peak early (E) and late (A) diastolic velocities, and E/A ratio were measured. Finally, by pulsed tissue Doppler, peak early diastolic velocity on the septal part of the mitral annulus was measured (Em) and E/Em ratio was calculated as previously described [20].

### Statistical analysis

Continuous variables were presented as mean and standard deviation, with independent t test used to compare differences between groups. Categorical variables were presented as counts and percentages, with Fisher’s exact tests used to compare differences between groups. Logistic regression was performed to detect which factor provides the strongest influence among myocardial fibrosis groups. Factors that were significant in univariate logistic regression were included in multivariate logistic regression, with stepwise model selection used to select the best factor to detect myocardial fibrosis. The receiver operating characteristic (ROC) curve was used to examine the factor performance in fibrosis and non-fibrosis groups. The cut-off value of factors that may distinguish myocardial fibrosis or not were selected using Youden index, which was defined as sensitivity + specificity − 1. Area under ROC curve with null hypothesis: AUC = 0.5 was performed using the Wilcoxon rank sum test. All statistical analyses were performed with SPSS software version 17 (SPSS Inc, Chicago, IL, USA), and two-tailed *p* < 0.05 indicated statistical significance.

## Results

### Comparison of baseline characteristics in HCM and control groups

Patients’ baseline characteristics in HCM and control groups are presented in Table 1. Among 122 subjects enrolled in this study, 76 were HCM patients and 46 were healthy subjects, including 82 males and 40 females with a mean age of 46.1 years. Mean heart rate was 70.5 beats/min, mean BSA was 1.7 m^2^, mean BMI was 23. 9 kg/m^2^, mean SBP was 122.5 mmHg andmean DBP was 80.4 mmHg. In the HCM group, 26.3 % had a history of HCM and 13.2 % had unexplained syncope.

**Table 1:** Baseline characteristics of patients in HCM and control groups

	HCM group (N = 76)	Control group (N = 46)	*p* value
Age(years)	47.1 ± 12.6	44.3 ± 6.9	0.116
Gender			0.667
Male	50 (65.8 %)	32 (69.6 %)	
Female	26 (34.2 %)	14 (30.4 %)	
Heart rate (beats/min)	70.5 ± 9.4	70.4 ± 2.5	0.982
BSA (m^2^)	1.7 ± 0.2	1.7 ± 0.1	0.707
BMI (kg/m^2^)	24.1 ± 2.0	23.5 ± 1.5	0.101
SBP (mmHg)	124.3 ± 6	119.5 ± 7.1	<0.001
DBP (mmHg)	81.8 ± 5.8	78 ± 5.1	<0.001
Family history of HCM	20 (26.3 %)	0 (0 %)	<0.001
Unexplained syncope	10 (13.2 %)	0 (0 %)	<0.001

No significant differences were found between the HCM and control group in age, gender, heart rate, BSA and BMI (*p* > 0.05). The means of SBP and DBP were significantly higher in the HCM group than in the control group (SBP: 124.3 vs. 119.5, *p* < 0.001; DBP: 81.8 vs. 78, *p* < 0.001). The HCM group had more patients with a history of HCM and unexplained syncope than the control group (history of HCM: 20 vs. 0, *p* < 0.001; unexplained syncope: 10 vs. 0, *p* < 0.001) (Table 1).

### Comparison of echocardiography parameters in HCM and control groups

**Fig. 1:**
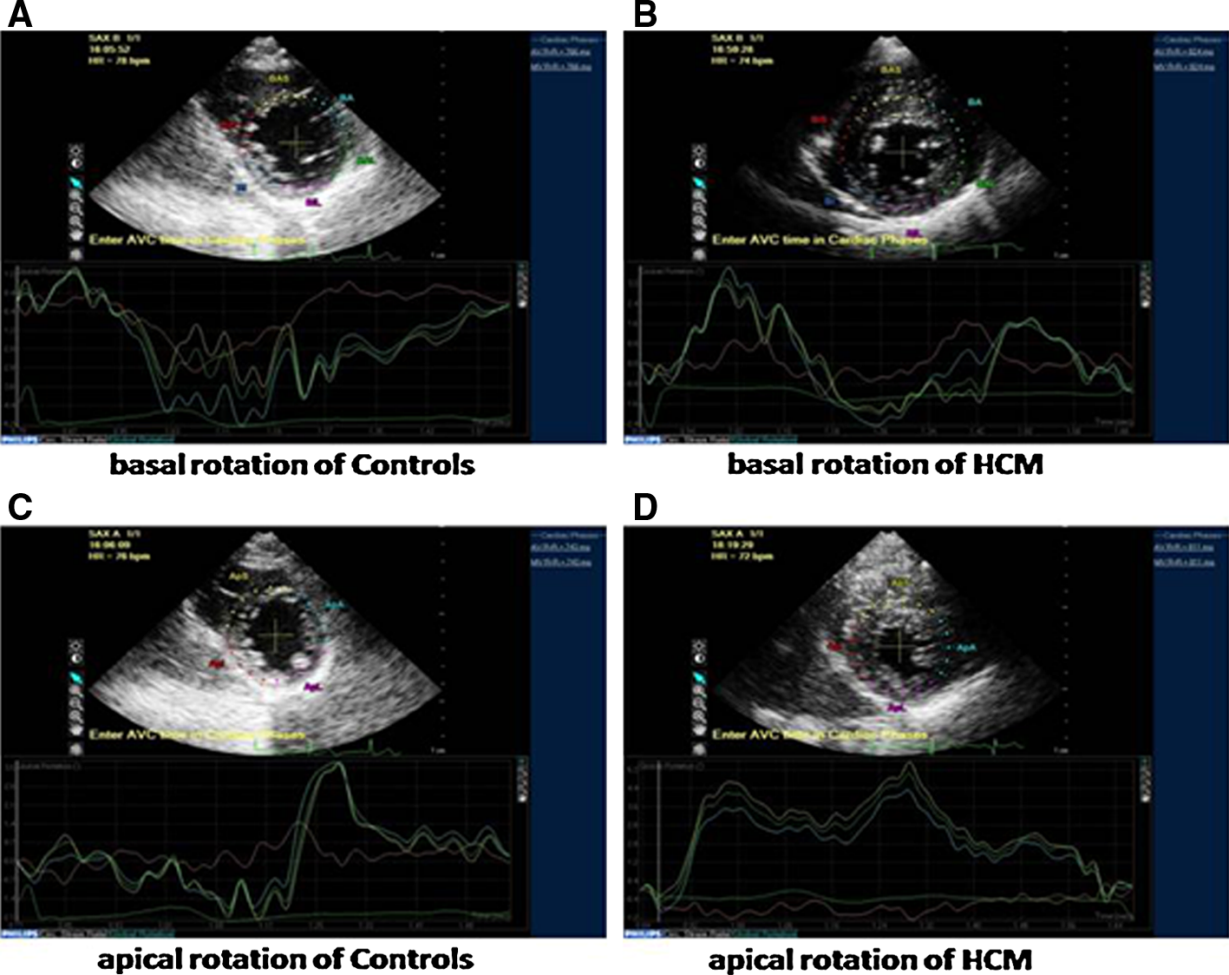
Two-dimensional speckle tracking images in the short-axis view at the mitral valve and apical level are shown. Apical rotation, basal rotation, and LV twist in HCM patients is significantly higher than in controls. *HCM* hypertrophic cardiomyopathy

Comparisons of echocardiography parameters between HCM and control groups are presented in Table 2. Among 122 patients, the mean Bas-Rotation was 7.4°, mean AP-Rotation was 9.9°, mean LV-Twist was 17.3°, mean LVEF was 69.2 %, mean LADs was 37.4 mm, mean LVDd was 42.3 mm, mean IVST was 16.3 mm, mean LAVi was 43.5 ml/m^2^, mean E/A was 1.1, mean E/Em was 12.3 and mean LVMI was 100.2 g/m^2^. The HCM group had significantly higher values for Bas-Rotation, AP-Rotation, LV-Twist, LVEF, LADs, LAVi, IVST, LVPWT, RWT, E/Em and LVMI than the control group (all *p* < 0.0001). LVDd and E/A were significantly lower in the HCM group than in the control group (both *p* < 0.001) (Table 2). Representative examples are shown in Fig. 1.

**Table 2:** Comparison of echocardiographic parameters in HCM and control groups

	HCM (N = 76)	Control (N = 46)	*p* value
Bas-Rotation (°)	8.6 ± 1.8	5.5 ± 0.7	<0.001
AP-Rotation (°)	11.2 ± 2.4	7.8 ± 0.6	<0.001
LV-Twist (°)	19.8 ± 4.0	13.2 ± 0.9	<0.001
LVEF (%)	70.4 ± 6.0	67.3 ± 3.8	0.001
LADs (mm)	40.6 ± 3.1	32.1 ± 2.0	<0.001
LVDd (mm)	41.0 ± 2.1	44.5 ± 1.5	<0.001
LAVi (ml/m^2^)	51.3 ± 5.4	30.7 ± 2.9	<0.001
IVST (mm)	20.7 ± 3.0	9.0 ± 0.6	<0.001
LVPWT (mm)	10.9 ± 0.8	8.8 ± 0.4	<0.001
RWT	0.5 ± 0.04	0.3 ± 0.01	<0.001
E/A	1.0 ± 0.3	1.3 ± 0.1	<0.001
E/Em	14.6 ± 3.2	8.5 ± 1.1	<0.001
LVMI (g/m^2^)	119.1 ± 22.5	68.9 ± 7.6	<0.001
GLS	−14.2 ± 1.8	−18.0 ± 0.4	<0.001
Untwisting velocity	−10.5 ± 1.1	−13.1 ± 0.4	<0.001

### Distribution of characteristics in HCM patients with and without fibrosis

**Fig. 2:**
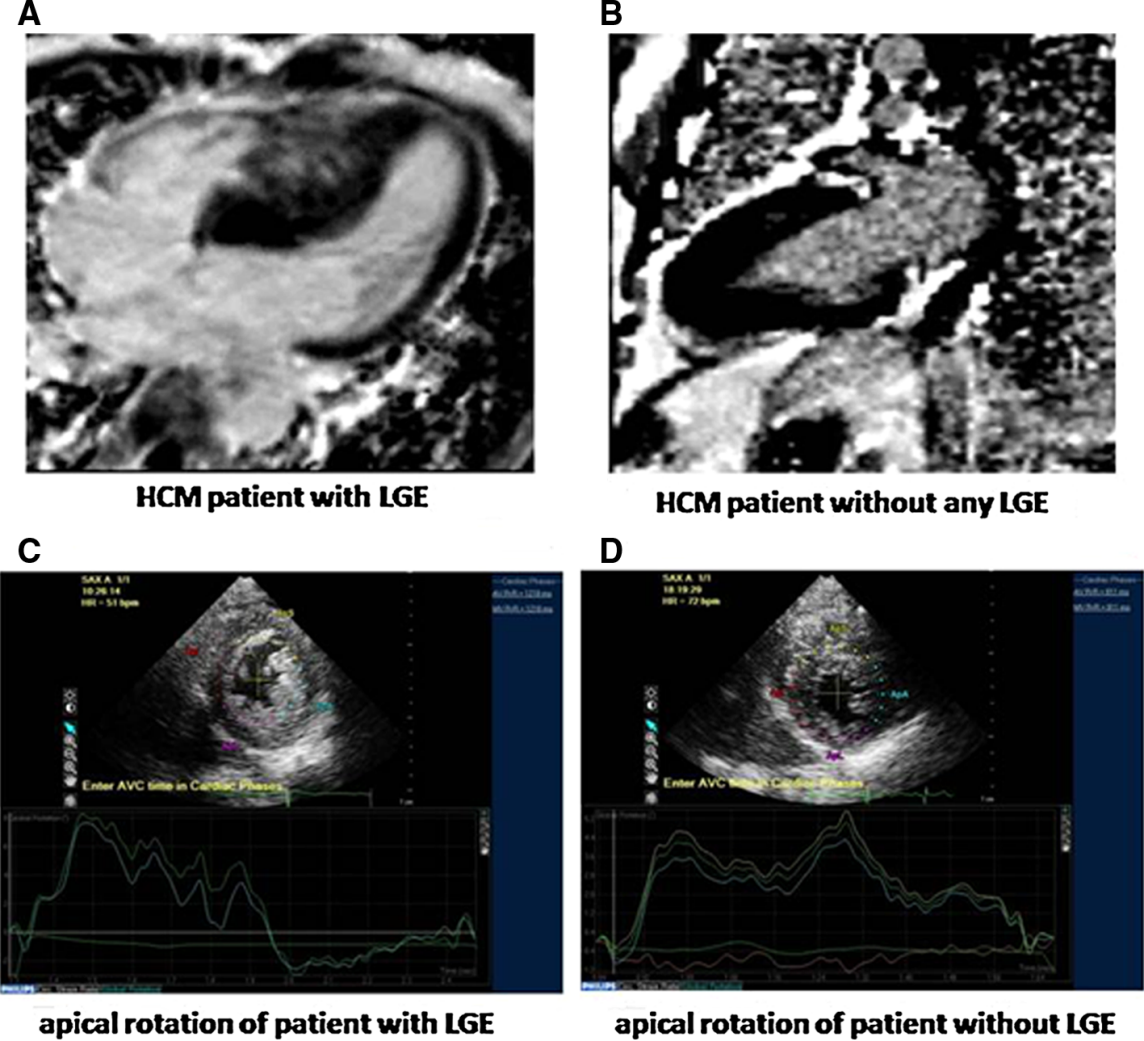
Images are shown for an HCM patient with large LGE (**a**) and a patient without LGE (**b**). LV twist in the patient with LGE(**c**) was higher than that in the patient without LGE (**d**). *HCM* hypertrophic cardiomyopathy, *LGE* late gadolinium enhancement, *LV* left ventricular

The distribution of characteristics in HCM patients with and without fibrosis is presented in Table 3. In the 76 HCM patients, means for Bas-Rotation, AP-Rotation, LV-Twist, LADs, LAVi, IVST, LVPWT, RWT, E/Em, and LVMI were all significantly higher in the fibrosis group than in the non-fibrosis group (*p* < 0.001). Means of age and E/A were significantly lower in the fibrosis group compared with non-fibrosis group (age: 44.6 vs. 51.7 years, *p* = 0.007; E/A: 1.0 vs. 1.2, *p* = 0.001). No significant differences were found between non-fibrosis vs. fibrosis groups in heart rate, BSA, BMI, SBP, DBP and LVEF (*p* > 0.05). Representative examples are shown in Fig. 2.

**Table 3:** Distribution of characteristics in HCM patients with and without fibrosis

	Non-fibrosis (N = 27)	Fibrosis group (N = 49)	*p* value
Age (years)	51.7 ± 8.5	44.6 ± 13.8	0.007*
Heart rate (beats/min)	70.7 ± 10.6	70.4 ± 8.9	0.889
BSA (m^2^)	1.7 ± 0.2	1.8 ± 0.1	0.129
BMI (kg/m^2^)	24.2 ± 1.6	24.0 ± 2.3	0.693
SBP (mmHg)	123.7 ± 5.9	124.6 ± 6.1	0.524
DBP (mmHg)	81.4 ± 5.5	82.1 ± 5.9	0.628
Bas-Rotation (°)	6.7 ± 0.9	9.6 ± 1.3	<0.001*
AP-Rotation (°)	8.3 ± 0.9	12.7 ± 1.2	<0.001*
LV-Twist (°)	15.0 ± 1.1	22.4 ± 2.1	<0.001*
LVEF (%)	70.7 ± 3.7	70.2 ± 6.9	0.698
LADs (mm)	38.7 ± 1.1	41.7 ± 3.3	<0.001*
LVDd (mm)	41.6 ± 1.9	40.7 ± 2.1	0.052
LAVi (ml/m^2^)	45.1 ± 2.4	54.7 ± 2.9	<0.001*
IVST (mm)	18.3 ± 2.2	22.1 ± 2.4	<0.001*
LVPWT (mm)	10.7 ± 0.7	11.1 ± 0.8	0.02*
RWT	0.5 ± 0.04	0.5 ± 0.02	<0.001*
E/A	1.2 ± 0.2	1.0 ± 0.2	0.001*
E/Em	11.1 ± 1.6	16.6 ± 1.9	<0.001*
LVMI (g/m^2^)	98.3 ± 10.5	130.6 ± 18.8	<0.001*
GLS	−16.4 ± 0.9	−13.0 ± 0.6	<0.001*
Untwisting velocity	−11.4 ± 1.1	−10.0 ± 0.8	<0.001*

* Significant differences between non-fibrosis versus fibrosis groups

In addition, LV-twist showed a significant, positive and linear correlation with IVST, LVPWT, RWT, LAVi and E/Em in HCM patients with fibrosis. The greater the increase in LV-twist, the greater were IVST, LVPWT, RWT, LAVi and E/Em. No significant linear correlations were found between LV-twist and E/A, untwisting velocity (Supplemental Table 2).

### Univariate and multivariate logistic regression analysis of effect factors for fibrosis in HCM patients

**Fig. 3:**
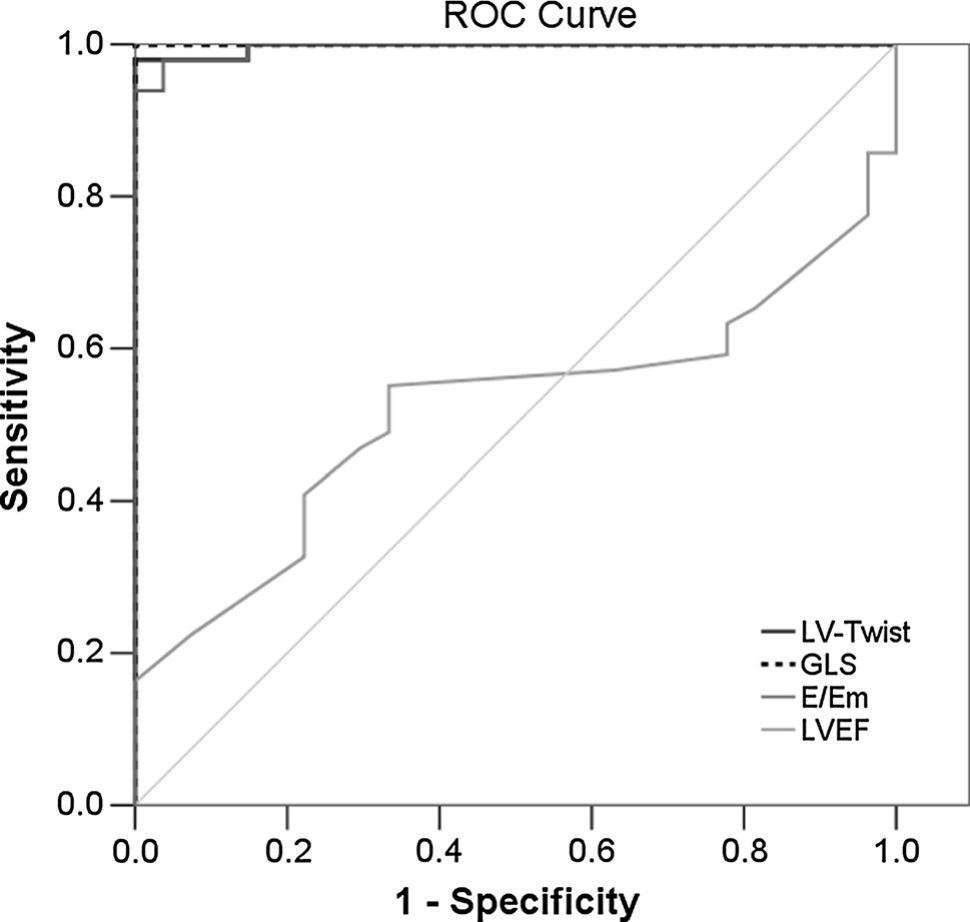
The receiver operating characteristic (ROC) curves for LV-twist, GLS, E/Em and LVEF in HCM patients with and without fibrosis. *GLS* global 2-dimensional strain

Univariate logistic regression revealed that the significant effect factors for fibrosis in HCM patients were age, Bas-Rotation, AP-Rotation, LV-Twist, LADs, IVST, LAVi, E/A, E/Em, LVMI, LVPWT and RWT. When these variables were recruited into multivariate logistic regression, stepwise model selection revealed that LV-twist would be the best factor to detect myocardial fibrosis (Supplemental Table 1). When ROC curve analysis was used to examine the LV-Twist performance in HCM patients with and without myocardial fibrosis, the AUC showed high discriminatory power for LV-twist to distinguish between myocardial fibrosis or no fibrosis (AUC 0.99, 95 % CI 0.99–1.0, *p* < 0.001, Fig. 3); with an optimal cut-off value of 18.5 for LV-Twist as the lower limit at which to detect patients with fibrosis. Youden index determined sensitivity and specificity to be 98 and 100 %, respectively. The AUC also showed high discriminatory power for GLS and E/Em to distinguish between myocardial fibrosis or no fibrosis (GLS: AUC 1.0, 95 % CI 1.0–1.0, *p* < 0.001; E/Em: AUC 0.99, 95 % CI 0.99–1.0, *p* < 0.001) (Fig. 3); with an optimal cut-off value of -14.7 for GLS and 13.4 for E/Em as the lower limit at which to detect patients with fibrosis. Youden index determined sensitivity and specificity to be 100 and 100 % for GLS, and 98 and 96.3 % for E/Em (Table 4).

**Table 4:** Optimal cutoff value of LV twist by Youden index summary of AUROC curve

	Optimal	Sensitivity	Specificity	AUC	*p* value
	Cutoff value	(95 % CI)	(95 % CI)	(95 % CI)	
LV-twist	≥18.5	98 % (87.8–99.9 %)	100 % (84.5–100 %)	0.99 (0.99–1.0)	<0.001
GLS	≥−14.7	100 % (92.7–100 %)	100 % (87.2–100 %)	1.0 (1.0–1.0)	<0.001
E/Em	≥13.4	98 % (89.1–99.9 %)	96.3 % (81.0–99.9 %)	0.99 (0.99–1.0)	<0.001
LVEF	≥70.5	55.1 % (40.2–69.3 %)	66.7 % (46.0–83.5 %)	0.5 (0.4–0.6)	0.78

*GLS* global two-dimensional strain

## Discussion

Results of the present study demonstrate that patients with HCM, even in the presence of preserved EF, show impaired regional myocardial deformation with increased LV twist. LV twist is significantly associated with myocardial fibrosis segments, suggesting that LV twist may provide useful information on the presence and extent of myocardial fibrosis and cardiac events in HCM patients with normal LVEF. Differences in diagnostic parameters between HCM patients and controls.

Our results showed that the apical horizontal rotation angle, base rotation angle and left ventricular torsion angle in HCM patients were significantly higher than those in healthy controls. This might be explained by the fact that cardiac hypertrophy in HCM patients may increase the moment of epicardial fibers, resulting in dominant counterclockwise rotation of the epicardial fibers. Miyazaki et al. [21] studied HCM patients at the molecular level, showing that the differential pressure across the walls due to the decrement of light chain-related phosphorylated myosin concentration may promote left ventricular rotation. The hypertrophic myocardium protrudes into the ventricular cavity, which reduces the left ventricular volume. Therefore, to maintain the normal ejection fraction, the ventricular contraction may increase as compensation, but the left ventricular contraction occurs by auto-rotation. Our results also showed that LV and EF, two parameters commonly used to evaluate the left ventricular contraction, were comparable between the HCM patients and controls. This suggests that the left ventricular contraction in HCM at the early stage is still compensatory, and traditional echocardiography usually fails to reflect early left ventricle dysfunction. However, STE may sensitively reflect the dysfunction of the left ventricle in patients with HCM at an early stage.

In another study, apical rotation, basal rotation and torsion or LV twist were increased in patients with HCM compared to results of these CMRI parameters in normal volunteers [22]. In that study, HCM patients examined by magnetic resonance tagging showed an increased degree of LV rotation. Owing to the after-load dependency of LV twist, this increased degree may be associated with increased mass/volume ratio, thus reduced afterload is characteristic of HCM patients. In the present study, while the difference in LVEF results between the HCM and control groups was not statistically significant, it is consistent with the results of Van Dalen et al. [23], who showed that apical rotation and twist (maximum) in HCM patients depend on patterns of LV hypertrophy. Thus, assessment of LV twist and rotation provides reliable quantitative evaluation of ventricular regional and global function and, as such, may be the more sensitive and specific indicator of subclinical myocardial dysfunction [24]. In general, patients with HCM have preserved LVEF as assessed by conventional methods. Serri et al. [25] reported that global 2D strain (GLS) was significantly reduced in HCM patients compared with normal control subjects, despite that the two groups had similar ejection fractions. Those authors concluded that STE can identify early abnormalities in HCM patients who have apparently normal LV ejection fractions. In the present study, GLS data (Table 4; Fig. 3) helps to explain that although HCM patients have normal LVEF, patients with myocardial fibrosis have lower GLS than those of healthy controls and HCM patients without myocardial fibrosis, which indicates the effectiveness of STE in predicting fibrosis and its prognostic value in HCM patients.

A review by Sengupta et al. [10] suggested that routine application of LV twist using the algorithm twist = mean peak apical rotation − mean peak basal rotation allows clinical differentiation of LV dysfunction found in day-to-day cardiology practice. Additionally, a recent case study by Tanaka et al. [26] concluded that 3-D speckle-tracking strain provides a reliable evaluation of and accurate information about true LV mechanics, identifying LV dyssynchrony using pyramidal 3-D data sets acquired in the same beats, which overcomes the limitations of 2D speckle-tracking radial strain and 2D tomographic imaging planes.

### Differences between HCM patients with and without myocardial fibrosis

In the present study, left ventricular myocardial fibrosis was detected in 49 HCM patients (65.5 %), and LV twist and apical rotation were significantly higher in the fibrosis subgroup than in the non-fibrosis subgroup. This may be the result of subendocardial ischaemia with dysfunction of subendocardial fibres causing rotation of the apex in a clockwise direction [15]. Microvascular myocardial ischemia, abnormal calcium handling and mechanical dyssynchrony are other possible mechanisms that could explain abnormalities of active relaxation, while fibrosis and myocardial hypertrophy may increase passive LV stiffness [27]. In addition, results of that study showed that the amount of segmental LGE correlated strongly with segmental wall thickening measured by MRI. Similarly, the present study showed that peak LV twist in fibrosis subgroups was significantly higher than in non-fibrosis subgroups, which suggests that LV twist, as well as LV rotation, is also affected by the extent of myocardial fibrosis.

We also found differences between groups in the left ventricular torsion angle, which was significantly larger in the fibrosis group than in the non-fibrosis group. This may be ascribed to subendocardial myocardial hypoperfusion due to myocardial fibrosis, affecting endomyocardial function and reducing negative torsional deformation. In addition, the apical horizontal rotation angle was markedly larger than that in the non-fibrosis group, but the base rotation angle was comparable between the two groups. Thus, the overall left ventricular rotation angle in the fibrosis group increased significantly compared to that in the non-fibrosis patients. Popovic et al. [28] applied STE to analyze the relationship between the left ventricular longitudinal strain and the number of myocardial fibrotic segments. Their results showed that the left ventricular longitudinal strain was negatively associated with the number of myocardial fibrotic segments. This suggests that the larger the area of myocardial fibrosis, the smaller the left ventricular longitudinal strain. In addition, our findings also revealed that the ventricular septum in the fibrosis group was thicker than that in non-fibrosis group. It appears that the thicker the ventricular wall, the more severe is the ischemia of myocardial microcirculation; in turn, myocardial microcirculation ischemia may induce myocyte apoptosis and deposition of collagens, which facilitates myocardial fibrosis.

In the present study, the E/Em and left atrial volume index in the fibrosis group were significantly higher than those in the non-fibrosis group. This may be ascribed to the myocardial interstitial fibrosis and the subsequent increase in ventricular wall stiffness and ventricular wall tension. In addition, the reduction in myocardial relaxation capability and compliance may have resulted in restricted ventricular filling in the early diastolic phase, with an increase in left atrial pressure and subsequent elevation in left atrial volume and E/Em. Additionally, the anteroposterior diameter in the left atrial systolic phase was comparable between the fibrosis group and non-fibrosis group, but marked difference was observed in left atrial volume index. This implies that the frequently used left atrial anteroposterior diameter may not accurately reflect the actual size of the left atrium.

Results of the present study revealed no differences in LVEF between the two subgroups. However, we observed that patients with fibrosis demonstrated larger left atrial volumes, LAVi and diastolic dysfunction as measured by E/A-ratio and tissue Doppler-derived methods (i.e., E/Em-ratio) as early signs for LV pathology and LV diastolic dysfunction. These data agree with results of previous studies showing early onset of markers of diastolic dysfunction in HCM patients [21, 29]. As stated above, several other mechanisms are potential causes of this functional impairment, including microvascular myocardial ischemia, abnormal calcium handling and mechanical dyssynchrony [27]. Recently, Nagakura et al. [30] evaluated LV dyssynchrony in HCM patients, finding that although LVH is not always associated with LV dyssynchrony, greater reductions in regional strain and severe dyssynchrony may be responsible in part for the adverse outcomes frequently occurring in HCM. Findings from that study showed that even when global LV systolic function is preserved, LV dyssynchrony is characteristic in HCM; genesis is typically at the apex and is believed to be due to heterogeneous myocardial wall thickening and relaxation. In an earlier study [31], application of STE revealed that deterioration of LV strain and twist mechanics may be due to a high LV outflow gradient (LVOT), since LVOT peak velocity was associated with LV mid-rotation and LV untwist in HCM patients compared to normal controls. Such evidence suggests that myocardial fibrosis appears to contribute significantly to the progressive deterioration of LV diastolic function.

### Association between LV twist and the extent of myocardial fibrosis in HCM

In the present study, the left ventricular torsion angle was closely related to the number of myocardial fibrotic segments, suggesting that LV twist analysis has good sensitivity and specificity in reflecting the severity of myocardial fibrosis in HCM patients. The area under the ROC curve was 0.814 using LV twist to detect myocardial fibrosis (95 % CI 0.625–0.890), which indicates that LV twist can sensitively and specifically reflect the degree of myocardial fibrosis in HCM patients. Our results are consistent with those of Saito et al. [32], who demonstrated that 2D global strain could identify subclinical global systolic dysfunction even though LV chamber function was normal in HCM subjects. Those authors concluded that global 2D strain was an important parameter of myocardial fibrosis in HCM patients. The present study also showed that the cutoffpoint for predicting myocardial fibrosis using LV twist was 18.22°, showing it to be a convenient and rapid quantitative parameter for clinical treatment and prognosis. Essentially, the larger the left ventricular torsion angle, the more severe is the myocardial fibrosis in HCM patients.

### Limitations

The present study has several limitations. First, the number of patients in the study was small, which results in relatively weak statistical power. Secondly, LV twist measured by 2D speckle tracking was not actually global twist because LV twist was measured from only three short-axis views (at the mitral valve and apical level). Since the distribution of LV twist and hypertrophy in the left ventricular is not uniform in HCM, it is possible that LV twist did not completely reflect the extent of LV myocardial fibrosis. The further development of three-dimensional (3D) speckle tracking might resolve this issue, as suggested by other authors [33]. Thirdly, the acquisition of orbicular LV short-axis images from base to apex is often technically difficult and may be settled by continued advances in 3D speckle tracking as previously suggested [34]. Fourthly, all the systolic parameters (apical rotation, basal rotation, LV twist) were heart-rate-independent because we measured peak values and did not measure timing. Finally, the ROI width at the hypertrophic segment might not cover the entire wall, because we adjusted the ROI to the thinner side of the myocardial wall. However, in the present study, LV twist did not depend on the LGE location in the myocardial wall. The development of software that can precisely trace the epicardial border might resolve this issue [33]. More clinical studies with larger patient populations are needed to confirm the validity of LV twist as a marker for myocardial fibrosis and as a predictor of major cardiac events. Future studies with larger sample-size will include patient follow-up to further confirm the relationship between left ventricular torsion angle and the severity of myocardial fibrosis. We would aim to elucidate whether the left ventricular torsion angle may be used to evaluate myocardial fibrosis or is independent of myocardial fibrosis, and whether it may predict prognosis for HCM patients. If so, LV twist analysis may serve as a simple, convenient indicator for clinical risk stratification and evaluation of therapeutic efficacy.

## Conclusions

In conclusion, LV twist and rotation in patients with HCM can be accurately measured using STE and results may be relevant to making clinical decisions. LV twist evaluation may provide useful information on myocardial fibrosis and cardiac events in HCM patients with normal chamber function. This clinically promising parameter may be useful for risk stratification in patients with HCM.

## Electronic Supplementary Material

Below is the link to the electronic supplementary material.

Supplementary material 1 (DOC 30 kb)
