# Supplementary material for: Assessment of left ventricular twist mechanics by speckle tracking echocardiography reveals association between LV twist and myocardial fibrosis in patients with hypertrophic cardiomyopathy
Source: Int J Cardiovasc Imaging. 2014 Aug 9;30(8):1539–48. doi: 10.1007/s10554-014-0509-6 (PMC4232740; doi:10.1007/s10554-014-0509-6)
Supplement: Supplementary file 1 — Supplementary material 1 (DOC 30 kb) [file 10554_2014_509_MOESM1_ESM.doc]

Supplemental Table 1. Final result of multivariate logistic analysis according to stepwise model selection

|  | OR (95% CI) | p-value |
| --- | --- | --- |
| LV-twist | 7.15(1.67-30.6) | 0.008 |

Supplemental Table 2. Correlations between LV-twist and variables of wall thickness and diastolic impairment in HCM fibrosis patients

|  | r | p-value |
| --- | --- | --- |
| IVST | 0.38 | 0.007 |
| LVPWT | 0.35 | 0.013 |
| RWT | 0.32 | 0.028 |
| LAVi | 0.58 | <0.001 |
| E/A | -0.17 | 0.244 |
| E/Em | 0.43 | 0.002 |
| Untwisting velocity | 0.06 | 0.683 |
